# Unusual impulse-momentum relationship in non-reciprocal light interactions

**DOI:** 10.1038/s41377-025-02139-8

**Published:** 2026-02-09

**Authors:** Yuhui Zhuang, Juan Wu, Siyu Li, Yi Hu, Zhigang Chen, Jingjun Xu

**Affiliations:** 1 The MOE Key Laboratory of Weak-Light Nonlinear Photonics, TEDA Applied Institute and School of Physics, Nankai University, Tianjin, China; 2 Pengcheng Laboratory, Shenzhen, China

**Keywords:** Nonlinear optics, Nonlinear optics

## Abstract

Non-reciprocal interactions, featured with an asymmetric relation between action and reaction, underpin exotic phenomena across living and artificial systems. Albeit extensively studied, they have been largely underexplored in nonlinear interactions of waves. In this work, we report an unusual impulse-momentum relationship for an optical solitary wave whose internal interactions are non-reciprocal. The solitary wave gains either an enhanced or a reversed momentum relative to an impulse that is applied to one of its two components. In the regime where the solitary wave is not broken down, the impulse-momentum relationship is found to be linear, yet its slope is unusual - either exceeding one or even being negative. Our results may initiate more fundamental considerations related to non-reciprocal wave interactions that are useful for designing novel non-Hermitian devices.

**Figure:**
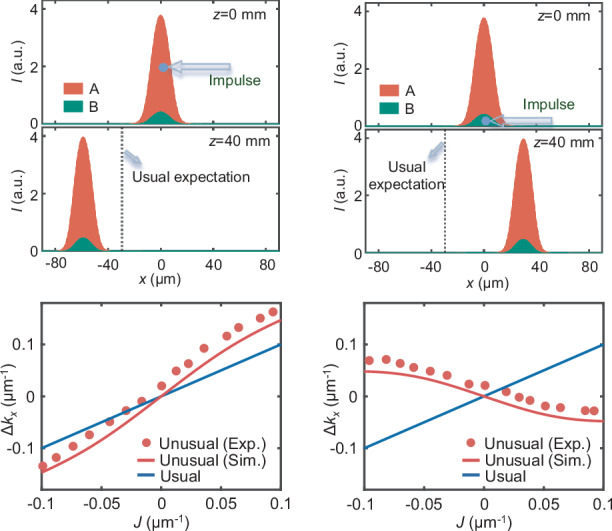
We report an unusual impulse-momentum relationship for an optical solitary wave whose internal interactions are non-reciprocal. An enhanced or even a reversed momentum compared to an impulse is gained.

We report an unusual impulse-momentum relationship for an optical solitary wave whose internal interactions are non-reciprocal. An enhanced or even a reversed momentum compared to an impulse is gained.

## Introduction

Non-reciprocal interaction is ubiquitous in nature. It is basically manifested as an asymmetric interplay between two agents, as widely seen in, for instance, predator-prey dynamics^1,2^, neuroscience^3,4^, pedestrian behavior^5,6^ and host-parasite relationship^7,8^. This interaction is generally in the framework of non-equilibrium dynamics, and it is crucial in the formation of collective and ordered motion^9–15^, which promotes the emergence of various life and ecological phenomena.

Inspired probably by nature, non-reciprocal interactions are also utilized to design novel artificial materials and devices^16–18^. Active matter is one of the most representative examples^19,20^. It can be synthesized by employing two species that have an asymmetric response to each other^21–24^, promising for generating self-organizing and intelligent functions^25–32^. Another example is to devise quantum systems^33^, in which the non-reciprocal interaction is generally embodied by an asymmetric coupling between two sites, giving rise to non-Hermitian features. Such a design has been extensively employed in the fields of spintronics^34–37^, photonics^38–44^, electronics^45–47^, mechanics^48–51^ and ultracold atoms^52,53^, leading to exotic phenomena represented by non-Hermitian skin effect^38–50,52–55^, continuous time crystals^56,57^ and nonreciprocal quantum phase transitions^37^, which have no counterparts in devices based on symmetric couplings. It should be noted that the non-reciprocal interactions differ from the behavior of non-reciprocal propagation of light^58^. The latter is associated with the breaking of time-reversal symmetry caused generally by the magneto-optical effect^59^, time-dependent modulation of material properties^60^, asymmetric nonlinear interaction^61^ and topological effects^62^.

Albeit the aforementioned significant advances, non-reciprocal interactions have been much less explored in nonlinear wave interactions that are of paramount importance in both fundamental and applicative aspects. Nonlinearity, manifested as a wave intensity dependent behavior, provides a tunable way to manipulate a system. It exists widely in Bose-Einstein condensates^63–68^, water waves^69,70^, plasma^71^ and light^72,73^, to mention a few. Yet studies exploring non-reciprocal wave interactions have long been rare, particularly in experiment. Quite recently, non-reciprocal nonlinear wave interactions were demonstrated based on an optical platform^74^. Owing to a design of a stroboscopic nonlinearity (belonging to a kind of competing nonlinearity^75–80^), the interaction of two optical waves becomes asymmetric, having an attraction-repulsion type. Using this unique setup, wave dynamics similar to the chase-and-run motion of a predator and a prey was observed^81^. Non-reciprocal interactions tend to induce additional momentum, thereby potentially altering the impulse-momentum relationship - a phenomenon that, to our best knowledge, has not been investigated previously.

In this work, we report unusual impulse-momentum relationships in non-reciprocal wave interactions based on an optical platform. Our study is performed by testing a solitary wave consisting of two optical beams that experience competing nonlinearities. The solitary wave gains a larger momentum than an external impulse that is applied to its one component, while it has a reversed momentum against the impulse switched to the other component. The impulse-momentum relationships are linear in both cases providing that the solitary wave is not disintegrated, but the associated proportional coefficients, either exceeding 1 or becoming negative, are at odds with the usual value (i.e., one). These outcomes are closely related to the non-reciprocal interactions of the two components. Our experimental results reproduce well these unusual impulse-momentum relationships.

## Results

### Theoretical analysis

In optics, a momentum change of light is related to a beam tilt that plays the role of an impulse. Considering the usual impulse-momentum relationship, an impulse $J$ is formulated as:

1 $$J=\varDelta {k}_{x}=k\,\sin \,\theta$$

where $\varDelta {k}_{x}$ is the gained momentum in a transverse dimension (defined by *x*), $k$ is a wave vector and $\theta$ characterizes the angle of a given tilt with respect to a longitudinal direction (denoted by $z$). We then study the impulse-momentum relationship in non-reciprocal light interactions. A realistic model that consists of two coupled equations is employed^74^:

2a $$i\frac{\partial {\psi }_{{\rm{A}}}}{\partial z}=-\frac{1}{2k}\frac{{\partial }^{2}{\psi }_{{\rm{A}}}}{\partial {x}^{2}}-\frac{k}{{n}_{0}}\varDelta n{\psi }_{{\rm{A}}}$$

2b $$i\frac{\partial {\psi }_{{\rm{B}}}}{\partial z}=-\frac{1}{2k}\frac{{\partial }^{2}{\psi }_{{\rm{B}}}}{\partial {x}^{2}}-\frac{k}{{n}_{0}}\varDelta n{\psi }_{{\rm{B}}}$$

where ${\psi }_{{\rm{A}},{\rm{B}}}$ are complex amplitudes of two optical beams, and ${n}_{0}$ is a linear refractive index. The nonlinear index change has a form of $\varDelta n=\gamma {|{\psi }_{{\rm{A}}}|}^{2}/(1+{|{\psi }_{{\rm{A}}}|}^{2})-\gamma {|{\psi }_{{\rm{B}}}|}^{2}/(1+{|{\psi }_{{\rm{B}}}|}^{2})$ [$\gamma$ is a nonlinear coefficient, see supplementary materials (SM)], which allows beams A and B to feel self-focusing and -defocusing nonlinearities, respectively. The non-reciprocal light interactions are manifested as follows: beam A generates a waveguide effect to attract beam B, while the latter induces an anti-waveguide effect to repel the former. They are induced by a stroboscopic nonlinearity and occur transversely, which is quite different from the longitudinally non-reciprocal cases, manifesting as non-reciprocal light propagation that can be induced, for instance, by time modulations of the refractive index.

To explore the impulse-momentum relationship in the system, we consider an ideal scenario where the two beams form a stationary state manifested as a solitary wave, rather than the unconfined configuration shown in Ref.^74^. To obtain this solution, we set ${\psi }_{{\rm{A}}}={u}_{{\rm{A}}}(x)\exp (i{\beta }_{{\rm{A}}}z)$ and ${\psi }_{{\rm{B}}}={u}_{{\rm{B}}}(x)\exp (i{\beta }_{{\rm{B}}}z)$, where ${u}_{{\rm{A}},{\rm{B}}}(x)$ are real and ${\beta }_{{\rm{A}},{\rm{B}}}$ are eigenvalues (or propagation constants). Then one can readily obtain:

3a $$\frac{1}{2k}\frac{{\partial }^{2}{u}_{{\rm{A}}}}{\partial {x}^{2}}-{\beta }_{{\rm{A}}}{u}_{{\rm{A}}}+\frac{k}{{n}_{0}}\gamma \left(\frac{{|{u}_{{\rm{A}}}|}^{2}}{1+{|{u}_{{\rm{A}}}|}^{2}}-\frac{{|{u}_{{\rm{B}}}|}^{2}}{1+{|{u}_{{\rm{B}}}|}^{2}}\right){u}_{{\rm{A}}}=0$$

3b $$\frac{1}{2k}\frac{{\partial }^{2}{u}_{{\rm{B}}}}{\partial {x}^{2}}-{\beta }_{{\rm{B}}}{u}_{{\rm{B}}}+\frac{k}{{n}_{0}}\gamma \left(\frac{{|{u}_{{\rm{A}}}|}^{2}}{1+{|{u}_{{\rm{A}}}|}^{2}}-\frac{{|{u}_{{\rm{B}}}|}^{2}}{1+{|{u}_{{\rm{B}}}|}^{2}}\right){u}_{{\rm{B}}}=0$$

**Fig. 1:**
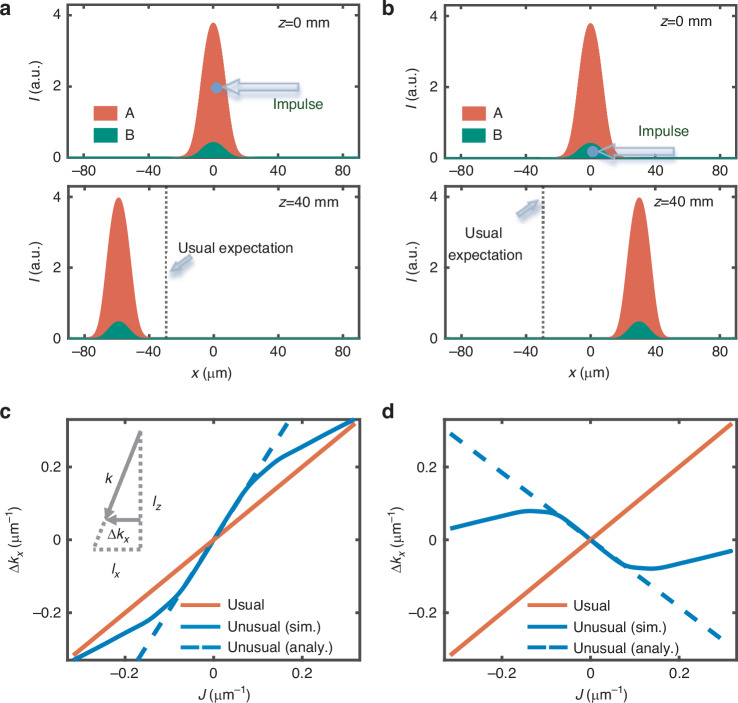
Unusual reaction of an optical solitary wave to an impulse. Left and right columns show the cases of applying an impulse to beam A and B, respectively. **a**, **b** Input and output profiles of the solitary wave for an impulse of $J=-0.02\,{\upmu}{{\rm{m}}}^{-1}$. Black dotted lines denote lateral beam shifts expected by the usual impulse-momentum relationship. **c**, **d** present simulated momentum changes for different impulses after a 40-mm-long propagation, as shown by blue solid curves. Blue dashed and red solid lines show the analytic results for the unusual and usual impulse-momentum relationships, respectively

For a solitary solution, the net index changes accumulated by the two inverted nonlinear processes should form a waveguide, and in turn, the two optical fields are exactly linear modes of the waveguide with ${\beta }_{{\rm{A}},{\rm{B}}}$ as the eigenvalues. Let us consider a simple configuration in which the two fields share the same mode of the nonlinearly-induced waveguide. Then their eigenvalues are equal to each other. A typical solution is presented in the upper panel of Fig. 1a, and its shape-invariant propagation is presented in the SM. Beam A has a stronger intensity to guarantee the self-trapping of the solitary wave. We then exert an impulse (i.e., a beam tilt) on the solution. In order to meet the paraxial condition shown in the propagation equations [i.e., Eq. (2)], a small tilting angle, meeting $\sin \,\theta \approx \theta$, is employed. When an impulse (towards left) acts on beam A only, the solitary wave shows a left shift that is larger than the value expected by the usual impulse-momentum relationship [see the bottom panel of Fig. 1a], indicating an additional momentum increment. For the other case (i.e., the same impulse is given to beam B), the solitary wave moves surprisingly towards right [Fig. 1b]. Figures 1c, d summarize the momentum changes for the solitary wave with different impulses. Under the paraxial condition, the momentum changes are calculated by $\varDelta {k}_{x}=k\frac{{l}_{x}}{{l}_{z}}$, where ${l}_{x}$ is the center-of-mass of a solitary wave after a propagation distance of ${l}_{z}$ [see the inset of Fig. 1c]. The resulting curves (solid blue) deviate from the usual impulse-momentum relationship (solid red). They are nearly linear in the range of $|J|\le 0.08\,{\upmu} {{\rm{m}}}^{-1}$, where the estimated proportional coefficients of them either exceed 1 or even become negative. Beyond this range, the impulse-momentum relationships are curved arising from the breakdown of the solitary wave upon a relatively large impulse.

**Fig. 2:**
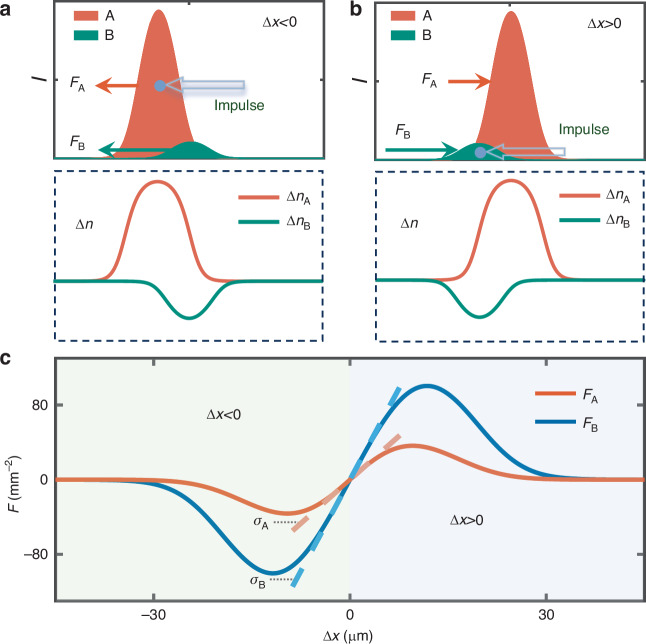
**Internal forces felt by the two beams composing a solitary wave**. **a**, **b** Force analysis (upper panels) for the solitary wave when an impulse is applied to one of its components: beam A (**a**) or B (**b**), and the related refractive index changes (bottom panels) induced nonlinearly by each component. **c** Values of the paired internal forces (solid lines) for different separations of the two beams. Dashed lines are tangential to the two curves in (c) at $\varDelta x=0$, and their slopes are represented by ${\sigma }_{{\rm{A}}}$ and ${\sigma }_{{\rm{B}}}$

Next, the origin of the unusual impulse-momentum relationships is investigated. We start with the case of tilting beam A only. As shown in Fig. 2a, beam A tends to move leftwards upon the impulse, while beam B (with no tilt) is lagged behind. Then lateral forces are triggered due to the non-overlapping of the two beams. They are induced by the nonlinearly-induced refractive index changes [bottom panel of Fig. 2a]. Beam A that feels the self-focusing nonlinearity induces a waveguide effect ($\varDelta {n}_{{\rm{A}}}$) to drag beam B towards left [see force ${F}_{{\rm{B}}}$ in Fig. 2a]; while beam B that experiences the self-defocusing nonlinearity produces an anti-waveguide effect ($\varDelta {n}_{{\rm{B}}}$) to repel beam A to the left [see force ${F}_{{\rm{A}}}$ in Fig. 2a]. Thus, the light interactions are able to introduce an additional momentum following the direction of the impulse. In the other tilting scenario (i.e., only beam B is given an impulse), the relative positions of the two beams tend to switch, and consequently, they feel forces against the impulse [Fig. 2b], which is why the solitary wave shifts laterally in the opposite direction of the impulse.

In order to carry out theoretical analysis, we consider a small impulse, upon which the solitary wave nearly maintains its shape during propagation. Then the forces felt by the two beams are assumed to be determined merely by the beam spacing. They are calculated by analyzing the potential (i.e.,$V=-\frac{k}{{n}_{0}}\varDelta n$) in Eq. (2), manifested as expectation values:

4a $${F}_{{\rm{A}}}=\frac{\langle {\psi }_{{\rm{A}}}|-\frac{\partial V}{\partial x}|{\psi }_{{\rm{A}}}\rangle }{\langle {\psi }_{{\rm{A}}}|{\psi }_{{\rm{A}}}\rangle }$$

4b $${F}_{{\rm{B}}}=\frac{\langle {\psi }_{{\rm{B}}}|-\frac{\partial V}{\partial x}|{\psi }_{{\rm{B}}}\rangle }{\langle {\psi }_{{\rm{B}}}|{\psi }_{{\rm{B}}}\rangle }$$

Figure 2c summarizes the forces as a function of beam separation (defined by $\varDelta x={x}_{{\rm{A}}}-{x}_{{\rm{B}}}$, where ${x}_{{\rm{A}}}$ and ${x}_{{\rm{B}}}$ are center-of-mass of the two beams). Keeping in mind the condition of a small impulse, we consider the zero limit of the spacing, where the interaction forces are approximately proportional to $\varDelta x$, i.e., ${F}_{{\rm{A}}}={\sigma }_{{\rm{A}}}\varDelta x$ and ${F}_{B}={\sigma }_{B}\varDelta x$ (${\sigma }_{{\rm{A}},{\rm{B}}}$ are parameters in analog to stiffness). Then the dynamics of the two beams (indeed equivalent to particles under the assumption of the profile invariance) can be analyzed by the following motion equations according to Ehrenfest theorem:

5a $${F}_{{\rm{A}}}={\sigma }_{A}({x}_{A}-{x}_{B})=k{\ddot{x}}_{{\rm{A}}}$$

5b $${F}_{{\rm{B}}}={\sigma }_{B}({x}_{A}-{x}_{B})=k{\ddot{x}}_{{\rm{B}}}$$

${\ddot{x}}_{{\rm{A}},{\rm{B}}}$ stand for $\frac{{d}^{2}{x}_{{\rm{A}},{\rm{B}}}}{d{z}^{2}}$. In the same vein, ${\dot{x}}_{{\rm{A}},{\rm{B}}}$ are $\frac{d{x}_{{\rm{A}},{\rm{B}}}}{dz}$, which are equal to tilting angles under the paraxial condition. For the case of tilting beam A only, one readily obtains ${x}_{{\rm{A}}}=-\frac{{\sigma }_{{\rm{A}}}\theta }{\omega ({\sigma }_{{\rm{B}}}-{\sigma }_{{\rm{A}}})}\sin (\omega z)+\frac{{\sigma }_{{\rm{B}}}\theta }{{\sigma }_{{\rm{B}}}-{\sigma }_{{\rm{A}}}}z$ and ${x}_{{\rm{B}}}=-\frac{{\sigma }_{{\rm{B}}}\theta }{\omega ({\sigma }_{{\rm{B}}}-{\sigma }_{{\rm{A}}})}\sin (\omega z)+\frac{{\sigma }_{{\rm{B}}}\theta }{{\sigma }_{{\rm{B}}}-{\sigma }_{{\rm{A}}}}z$, where $\omega =\sqrt{\frac{{\sigma }_{{\rm{B}}}-{\sigma }_{{\rm{A}}}}{k}}$, using the initial conditions of ${x}_{{\rm{A}}}=0$, ${x}_{{\rm{B}}}=0$, ${\dot{x}}_{{\rm{A}}}=\theta$, ${\dot{x}}_{{\rm{B}}}=0$ at $z=0$. After a sufficiently long distance (denoted by ${l}_{z}$), the first term (with a harmonic feature) of ${x}_{{\rm{A}}}$ or ${x}_{{\rm{B}}}$ is negligible as compared with the second term that linearly increases with the distance. Then both ${x}_{{\rm{A}}}$ and ${x}_{{\rm{B}}}$ are $\frac{{\sigma }_{{\rm{B}}}\theta }{{\sigma }_{{\rm{B}}}-{\sigma }_{{\rm{A}}}}{l}_{z}$, and so is the center-of-mass (i.e., ${l}_{x}$) of the solitary wave. Eventually, one can obtain an impulse-momentum relationship using the configuration shown in the inset of Fig. 1c:

6 $$\varDelta {k}_{x}=k\frac{{l}_{x}}{{l}_{z}}=\frac{{\sigma }_{{\rm{B}}}}{{\sigma }_{{\rm{B}}}-{\sigma }_{{\rm{A}}}}J$$

To derive the above formula, Eq. (1) is used under the paraxial condition (i.e., $\sin \,\theta \approx \theta$). Since ${\sigma }_{{\rm{B}}} > {\sigma }_{{\rm{A}}} > 0$ [as shown in Fig. 2c], $\frac{{\sigma }_{{\rm{B}}}}{{\sigma }_{{\rm{B}}}-{\sigma }_{{\rm{A}}}} > 1$. Using a similar way, one can obtain the impulse-momentum relationship for the case of tilting beam B only:

7 $$\varDelta {k}_{x}=-\frac{{\sigma }_{{\rm{A}}}}{{\sigma }_{{\rm{B}}}-{\sigma }_{{\rm{A}}}}J$$

which shows that a momentum change is minus proportional to an impulse. These analytical results [i.e., Eqs. (6) and (7)] have a good agreement with simulations for small impulses (i.e., $|J|\le 0.08\,{\upmu} {{\rm{m}}}^{-1}$) [Fig. 1c, d]. Beyond this range, they show deviations due to the invalidation of the wave invariance assumption. We should note that bound states that break the action-reaction symmetry can also be realized^82^ based on the counterintuitive dynamics of negative mass^83^, a mechanism different from that employed in our work. These states may yield distinct features in the impulse-momentum relationship.

### Experimental demonstrations

**Fig. 3:**
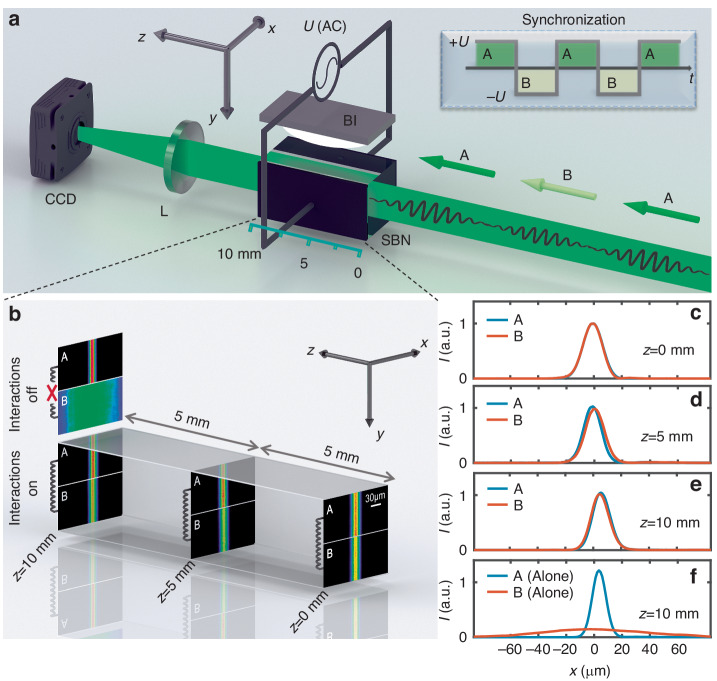
**Experimental realization of an optical solitary wave containing non-reciprocal internal interactions**. **a** Schematic setup: beams A and B, synchronized with positive and negative voltages applied on the SBN crystal, experience self-focusing and -defocusing nonlinearities, respectively, and they interact via the storage property of the crystal. **b** Measured intensity profiles of a solitary wave at the input and at the outputs after propagation distances of 5 mm and 10 mm. In the absence of the mutual interactions, the output profiles of its two components show typical self-focusing and -defocusing dynamics. **c**–**f** present the normalized beam profiles in (**b**) by integrating the intensity patterns over the *y*-axis

A schematic experimental setup used to measure impulse-momentum relationships in non-reciprocal light interactions is shown in Fig. 3a (see SM for more details). The desired optical nonlinearity is offered by a strontium barium niobate (SBN) crystal biased with an alternating current (AC) electric field. During the time of positive (negative) electric field, the nonlinearity is self-focusing (-defocusing). Two continuous-wave beams (labelled as A and B) chopped periodically are launched into the crystal along the *z*-axis in different time periods. The two beams have stripe shapes, nearly invariant along the *y*-axis, reducing the system to a simple configuration where only one transverse dimension (i.e., along the *x*-axis) is considered. Regarding the feature of the solitary solution, the two stripe beams employed in experiment are given Gaussian-like shapes. Beam A (or B), synchronizing with positive (or negative) electric fields, experiences self-focusing (or -defocusing) nonlinearity, as shown by its nonlinear output under the condition of switching off the other beam [Fig. 3b, f]. When they are both switched on, the two beams are able to interact thanks to the storage property of the crystal, albeit never meeting each other. By selecting appropriate input powers, the two beams are mutually confined to form a solitary wave with the assistance of white light as background illumination (BI), maintaining nearly unchanged profiles from the input to the output [Fig. 3b and Fig. 3c–e]. Owing to the stroboscopic nonlinearity, the solitary wave is a blinking steady-state (see SM).

**Fig. 4:**
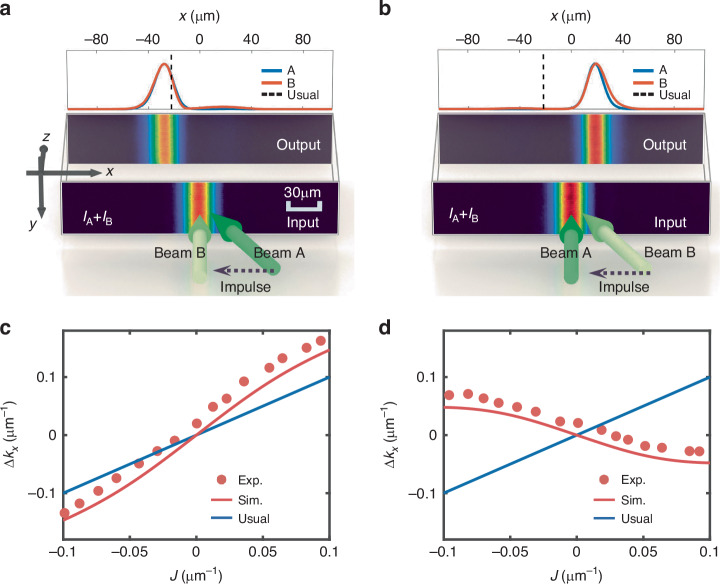
**Observed deflection of an optical solitary wave upon the action of** **an impulse**. **a,** **b** Schemes to apply an impulse, i.e., by tilting beam A (**a**) or B (**b**) only, and measured output profiles of the solitary wave (solid curves are obtained by integrating the intensity patterns vertically) (here, $J=-0.054\,{\upmu} {{\rm{m}}}^{-1}$). The dashed vertical lines show the output positions of the tilted beam only during a linear propagation, thus satisfying the usual impulse-momentum relationship. **c,** **d** Measured (dots) and simulated (red solid lines) momentum changes of the solitary wave for different impulses applied to beam A (**c**) or beam B (**d**). As a reference, blue solid lines show the usual impulse-momentum relationship

As performed in the simulations, we give an impulse to one of the two beams in the solitary wave, i.e., by introducing a beam tilt (see SM). Firstly, we tilt beam A to the left [Fig. 4a], which causes the solitary wave to move leftwards at the output. The resultant lateral shift of the solitary wave is larger than the case expected by the usual impulse-momentum relationship [vertical dashed line in Fig. 4a], indicating an extra increment of momentum. Secondly, the same impulse is applied to beam B, while beam A is injected normally [Fig. 4b]. In this case, the solitary wave exhibits a right lateral movement against the applied tilt. This counterintuitive behavior arises from the non-reciprocal interactions, as illustrated in Fig. 2b. The experimental results with other impulses are summarized in Fig. 4c, d, where the momentum change of the solitary wave is calculated by $\varDelta {k}_{x}=k\frac{{l}_{x}}{{l}_{z}}$ [see the inset in Fig. 1c]. In the chosen range of the impulse, the solitary wave can be maintained (see SM), thus the measured momentum change of the solitary wave is approximately proportional to the impulse, as indicated by the simulations in Fig. 1c, d. The proportional coefficient is estimated to be ~1.61 for the case of tilting beam A, exceeding the value of one in the usual impulse-momentum relationship; while its estimation is apparently negative (about -0.59) for the case of tilting beam B. The measured impulse-momentum relationship shows a slight upward shift compared to the simulations based on Eq. (2), which stems from the diffusion nonlinearity present in the experiment (see SM).

## Discussion

In conclusion, we have reported the unusual reactions of an optical solitary wave upon an external impulse. Such a solitary wave is composed by two components, one of which experiences a self-focusing nonlinearity and the other one feels a self-defocusing nonlinearity. The gained momentum for this entity is larger than the impulse that is exerted on the self-focusing component, while it is opposite to the impulse that is applied to the other component. These counterintuitive dynamics stem from the non-reciprocal internal interactions of the two components, leading to the generation of net momentum. Under the condition of a small impulse that does not break down the solitary wave, the momentum change is found to be proportional to the impulse, but the proportional coefficient, either exceeding one or appearing to be negative, is different from the case of the usual impulse-momentum relationship. These unusual relationships are further verified in experiment. Our work may trigger more fundamental studies based on non-reciprocal light interactions, which will potentially bring about novel applications associated with intelligent functions^84,85^.

## Supplementary information

Supplemental materials

## Data Availability

The data that support the findings of this study are available from the corresponding author upon reasonable request.
