# Supplementary material for: Unusual impulse-momentum relationship in non-reciprocal light interactions
Source: Light Sci Appl. 2026 Feb 9;15:111. doi: 10.1038/s41377-025-02139-8 (PMC12886815; doi:10.1038/s41377-025-02139-8)
Supplement: Supplementary file 1 — Supplemental materials [file 41377_2025_2139_MOESM1_ESM.docx]

Supplementary Materials for

**Unusual impulse-momentum relationship in non-reciprocal light interactions**

Yuhui Zhuang^1†^, Juan Wu^1, 2†^, Siyu Li^1†^, Yi Hu^1*^, Zhigang Chen^1^, Jingjun Xu^1††^

*^1^The MOE Key Laboratory of Weak-Light Nonlinear Photonics, TEDA Applied Institute and School of Physics, Nankai University, Tianjin 300457, China*

*^2^Pengcheng Laboratory, Shenzhen 518052, China*

^†^*These authors contribute equally to this work*

*yihu@nankai.edu.cn, ^††^jjxu@nankai.edu.cn

1) Theoretical parameters

For the experimental setting shown later on (i.e., an SBN crystal is applied with an AC voltage), the nonlinearity parameter $\gamma$ we use in simulations is estimated to be ~2.0×10^-4^ by using $\gamma=n_{0}^{3}\gamma_{33}E_{\text{ac}}/2$, in which $\gamma_{33}$ is the electro-optic coefficient along the crystal axis, $E_{\text{ac}}$ is the amplitude of the AC electric field and $n_{0}$ is the unperturbed extraordinary refractive index of the crystal, and their values are $\gamma_{33}=2.8\times10^{-10} \text{m} \text{V}^{-1}$, $E_{\text{ac}}=1.2\times10^{5} \text{V} \text{m}^{-1}$ and $n_{0}=2.3$.

2) Experimental setup

Our experimental setup is shown in Fig. S1 in more details. Through a polarizer and a half wave plate, the polarization direction of a laser is adjusted to be parallel to the optical axis of the crystal (along the *x*-axis) for gaining a large nonlinearity. Then the laser is divided into two beams: beams A and B. They are periodically switched on and off by optical shutters, i.e., OS1 and OS2, which are synchronized with positive and negative voltages in an AC voltage applied on the crystal, respectively. Consequently, beam A (B) is able to experience a self-focusing (-defocusing) nonlinearity in the SBN crystal. The frequency and amplitude of the AC voltage are 2 Hz and 600 V. A variable neutral density filter placed in the arm of beam B is used to alter the power ratio of the two beams. To generate the solitary wave, the powers of beams A and B are set to be 3.55 mW and 0.30 mW, respectively, and the crystal is illuminated by white light serving as background illumination. The two beams are focused at the front surface of the crystal by a cylindrical lens, and their input and output profiles are measured by a CCD assisted with a lens. An optical shutter (OS3, close to the CCD), synchronizing with either OS1 or OS2, allows us to selectively observe beam A or B, respectively. The tilt of beam A is performed by adjusting mirrors M1, M2 and M3, and the tilt of beam B is performed by adjusting mirrors M4, M5 and M6. The tilt of a beam can be obtained by the center-of-mass of the beam at the output ($x_{\text{out}}$), i.e., $\theta\approx\tan\theta=\frac{x_{\text{out}}}{l_{z}}$, where $l_{z}$ is the crystal length, after the setup is calibrated by using a reference beam that is launched normally and propagates linearly, corresponding to a tilt of $\theta=0$. Compared to the setup in Ref. [74], the one presented here has been updated: the variable neutral density filter has been added for generating the solitary wave, and controllable beam tilts (performed by mirrors M1-M6) have been introduced for observing the impulse-momentum relationship.


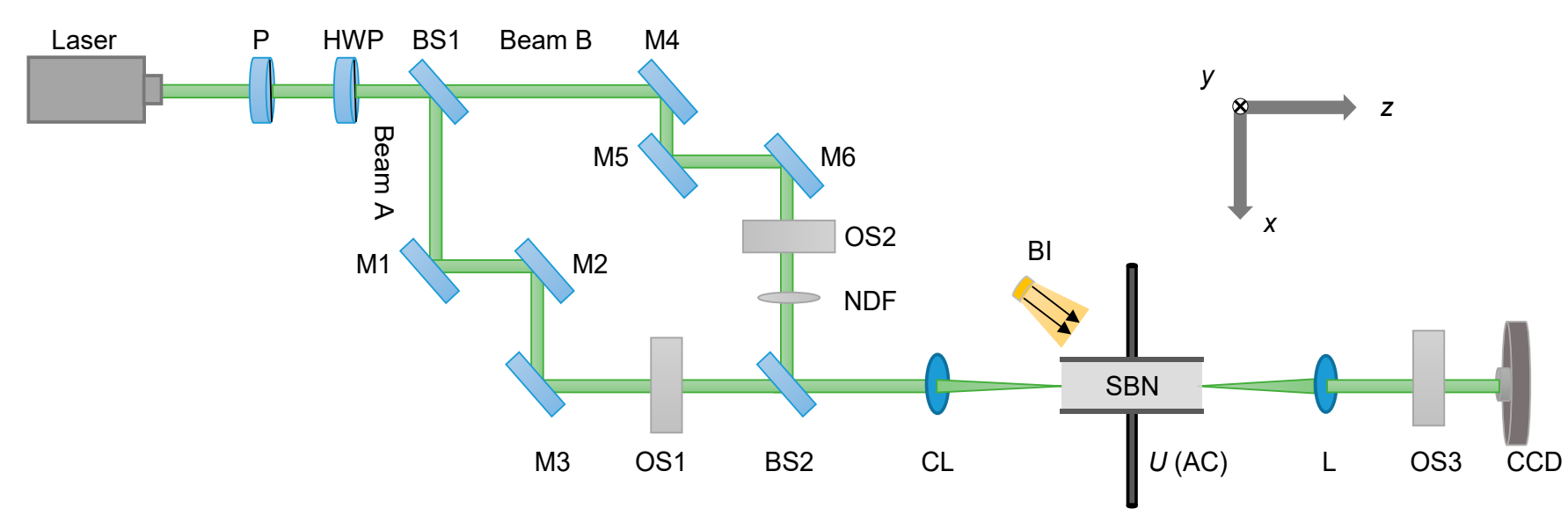


Fig. S1. Experimental setup. P, polarizer; HWP, half wave plate; BS, beam splitter; NDF, neutral density filter; CL, cylindrical lens; L, lens; CCD, charge coupled device; OS, optical shutter; M, mirror; BI, background illumination.

3) Propagation of a solitary wave

The shape-invariant propagation of the solitary wave shown in the upper panel of Fig. 1(a) is presented in Fig. S2.


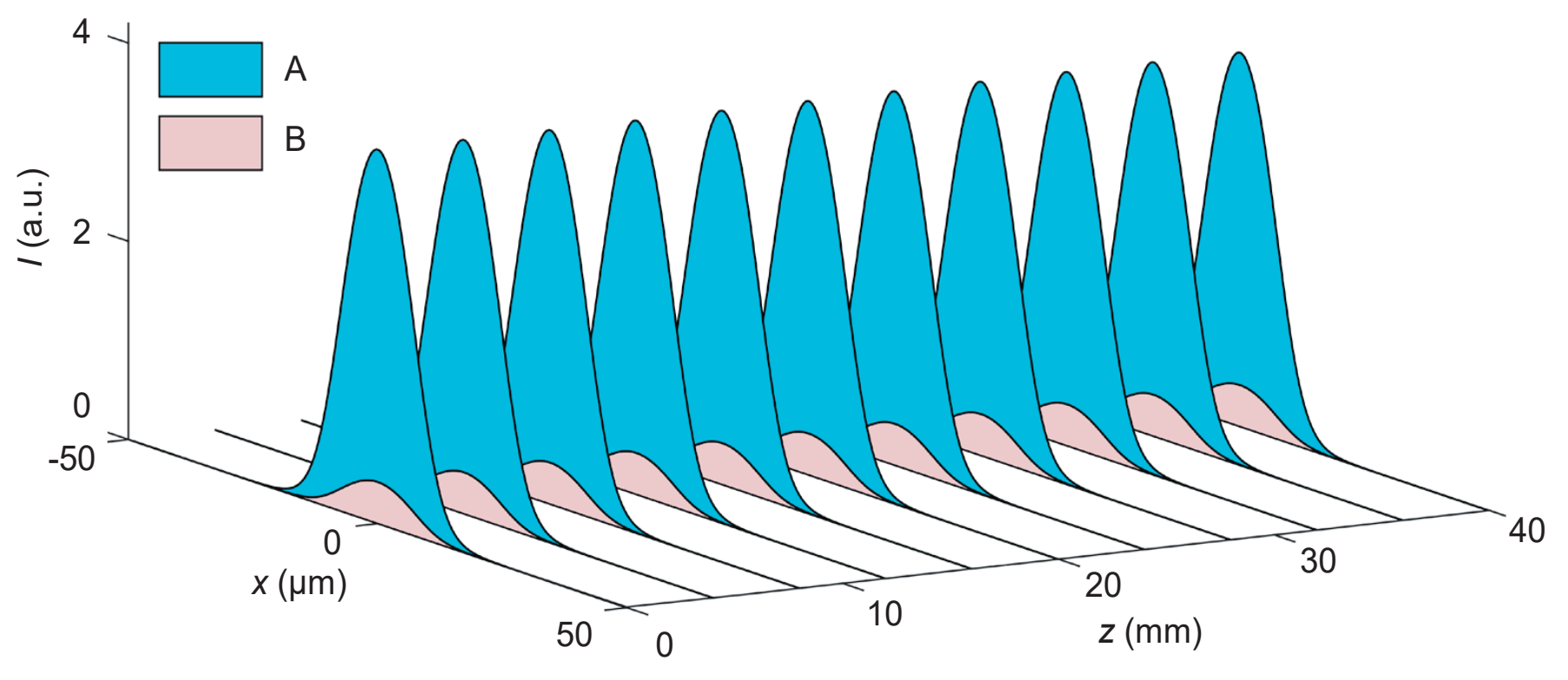


Fig. S2. Typical propagation of a solitary wave.

4) The steady state of the solitary wave

The solitary wave is blinking steady-state, rather than a transient phenomenon. As shown in Fig. S3, once formed, the solitary wave will maintain their shape unchanged over time.


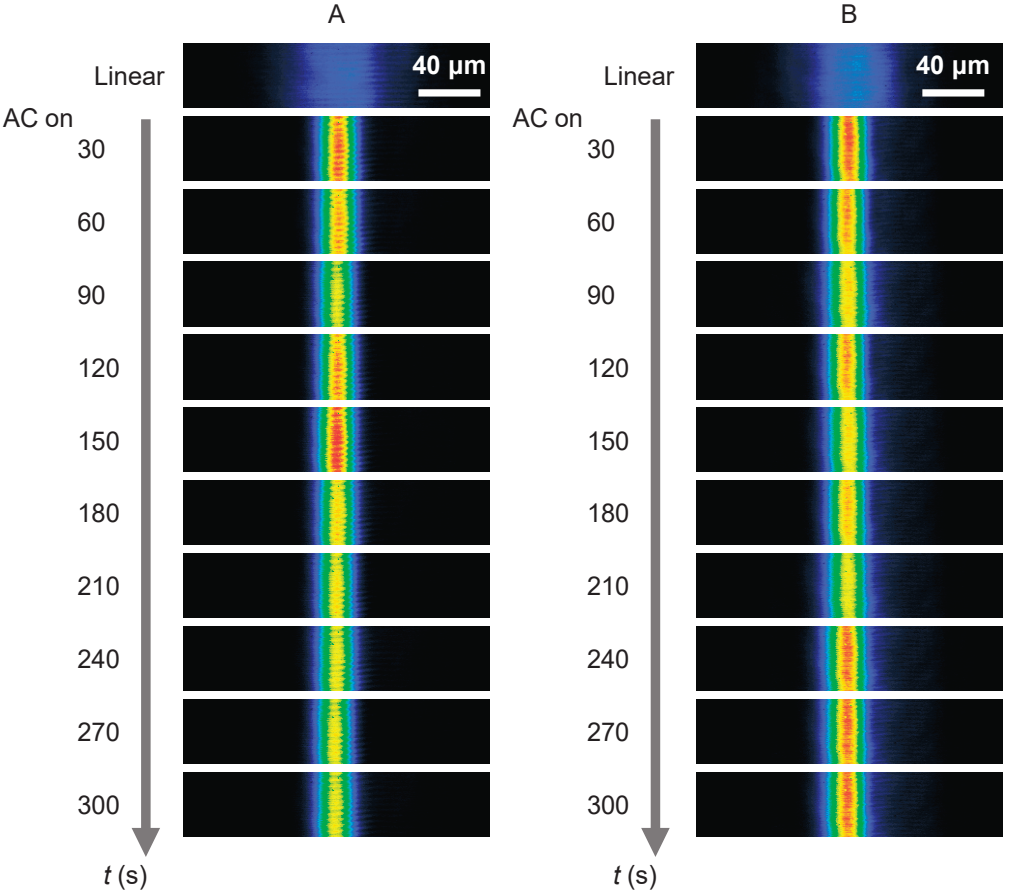


Fig. S3. Measured output profiles of the solitary wave at different time. First row: natural diffraction; other rows: solitary waves.

5) Regime where the solitary wave is maintained

Qualitatively, if its two components have localization similar to the input conditions, the solitary wave is maintained. In this regime, the two components can be approximately treated as particles and thus the resulting impulse-momentum relationship nearly matches the one obtained by the particle model [i.e., Eq. (5)], as shown in Figs. 1(c, d). In turn, one uses the matching region, i.e., $\left|J\right|\leq0.08 \mu\text{m}^{-1}$, to quantitatively identify the unbroken regime. In the experiment, we study the impulse-momentum relationship around this region. Figure S4 shows the measured outputs for various impulses. Apparently, their profiles have localization similar to the input ones.


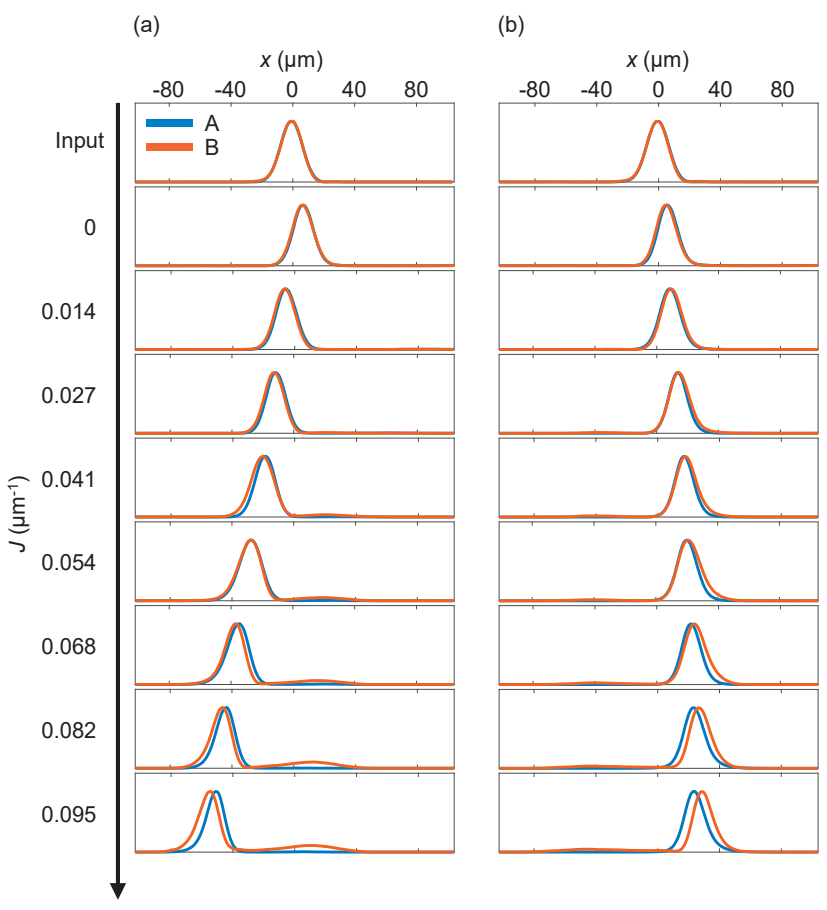


Fig. S4. Measured output profile for the case of applying an impulse to beam A (a) or B (b) only. The impulse increases from top to bottom. First row: input profiles; other rows: output profiles.

6) Influence of the diffusion nonlinearity (DNL)

The DNL is formulated as:

$\Delta n=\frac{1}{2}n_{0}^{3}\gamma_{33}\frac{K_{\text{B}}T}{e}\frac{1}{1+I}\frac{\partial I}{\partial x}$ (S1)

where $K_{\text{B}}$ is Boltzmann’s constant, $T$ is the absolute temperature, $e$ is the electron charge, $n_{0}$ is the unperturbed extraordinary refractive index of the crystal, and $\gamma_{33}$ is the electro-optic coefficient along the crystal axis. One can find that this type of nonlinearity is not dependent on the externally applied electric field. Thus, the self-bending caused by the DNL always points to the same direction in our setting, no matter which direction the electric field points to. To uncover the role of the self-bending effect, we perform simulations by using the experimental parameters and summarize the calculations in Fig. S5. In the absence of the DNL [corresponding to Eqs. (2a) and (2b)], the simulated impulse-momentum relationship slightly shifts downwards compared to the measurement. Once the DNL is added, the simulation matches well with experimental results. From this comparison, one can see that the DNL induces a shift of the impulse-momentum relationship, which stems from an additional momentum gain induced by the self-bending effect.


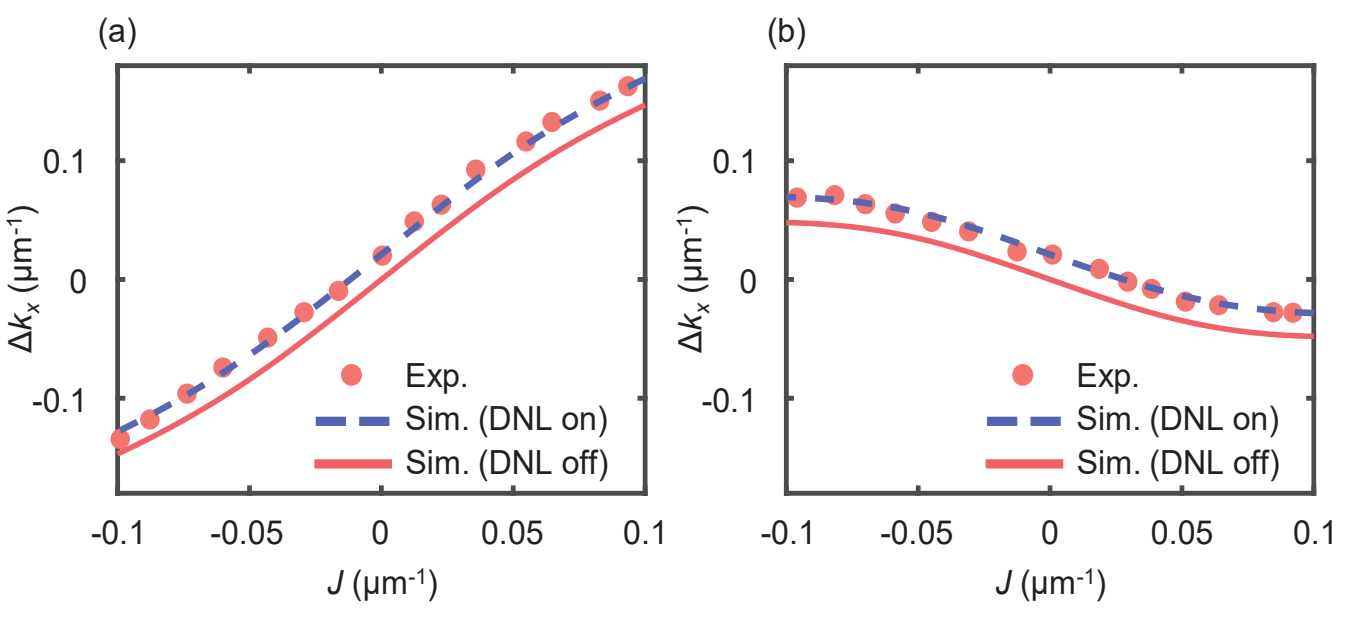


Fig. S5. Simulated impulse-momentum relationship with (dashed curves) and without (solid curves) the DNL, in comparison with the experimental results (dots). (a) or (b) corresponds to the kick of beam A or B only, respectively.
